# Sustainable and biocompatible Zn-based batteries

**DOI:** 10.1093/nsr/nwad055

**Published:** 2023-03-03

**Authors:** Guanjie He, Ivan P Parkin

**Affiliations:** Department of Chemical Engineering, University College London, UK; Christopher Ingold Laboratory, Department of Chemistry, University College London, UK; Christopher Ingold Laboratory, Department of Chemistry, University College London, UK

Next-generation and functional battery technologies beyond the Li-ion are emerging [1]. Sustainable and biocompatible batteries are needed for certain applications, including implantable medical devices [2]. Zinc-based batteries (ZBs) are encouraging technologies with low cost, high theoretical capacity and high safety features [3]. An increasing research interest has been attracted on developing ZBs to power flexible and wearable electronics [4]. Nevertheless, there was almost no research to consider the biocompatibility and biosecurity of ZBs.

Issues are existing such as limited energy density and poor stability of current ZBs. To make bio-integrated devices, the fabrication of each component in ZBs should be carefully considered, and the strict *in vivo* requirements should be met. The electrolyte is an essential component to realize biocompatible ZBs. Several candidates were proposed to improve the electrolyte performance, such as electrolyte additives, water-in-salt electrolytes, molecular crowding electrolytes, and polymer electrolytes [5]. Among them, polymer electrolytes will provide the programmability for biocompatible ZBs. However, challenges still need to be overcome including replacing toxic chemical reagents or initiators and generating green synthesis strategies.

In a recent study led by Prof. Jiang Zhou from Central South University, a novel Zn-alginate polymer electrolyte was prepared through an electro–cross-linking process without using any toxic chemical initiators or reagents [6]. Importantly, this polymer electrolyte can realize a high ionic conductivity through a well-aligned ionic pathway and adjustable thickness due to easily controlled electro–cross-linking time. As for an optimized fabrication, a uniform and dense polymer electrolyte was directly prepared on the Zn wire anodes within 80 s and formed a composite (i.e. Zn–Alg-5). To assemble full ZBs, MnO_2_ cathodes were twined onto the Zn–Alg-5 polymer electrolyte (Fig. 1a).

**Figure 1.**
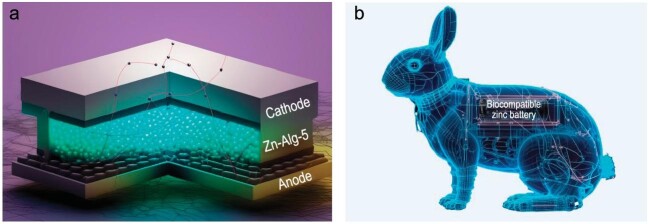
(a) The implantable zinc battery composed of a MnO_2_ cathode, a Zn wire anode, and the as-designed Zn–Alg-5 electrolyte. (b) Schematic diagram of biocompatible applications using rabbits as an illustration.

The as-fabricated ZBs exhibited excellent electrochemical performances, which were comparable to the ones in aqueous electrolytes. The full cells delivered a high-capacity retention, a good rate capability and an excellent stability. In addition, wire-shaped ZBs were fabricated in larger scales and the deformation (the change of bending angles) of ZBs had almost no influence on the battery performance. In this work, Zhou *et al.* also studied thoroughly the charge storage mechanism and excellent performance. A similar diffusion-controlled charge storage process of ZBs with Zn–Alg-5 electrolyte was observed compared with the ones in the liquid electrolyte. Especially, biosafety studies of the ZBs *in vivo* were conducted compared with typical Li, Na-ion batteries with organic electrolytes and ZBs with the aqueous electrolyte. All batteries were placed in the gastric mucosa of rabbits for 6 hours. Impressively, the smallest damaged area of the tissues was observed for ZBs with Zn–Alg-5, due to the inherently biocompatible materials in as-designed ZBs (Fig. 1b).

This study is a milestone to push forward the development of implantable and biocompatible ZBs. In the future, research can be focused on analysing the biocompatibility of ZBs for longer periods, and discussing the *in vivo* toxicity of different components, including cathodes, Zn anodes, electrolytes and packaging materials, in ZBs. More bio-friendly materials can be developed. In addition, the packaging and recycling of the biocompatible ZBs need to be systematically studied to further improve their sustainability.

## References

[1] Liu YY , Lu X, Lai FL et al. Joule 2021; 5: 2845–903. 10.1016/j.joule.2021.10.011

[2] Lei SZ , Liu ZX, Liu CX et al. Energy Environ Sci 2022; 15: 4911–27. 10.1039/D2EE02267B

[3] Zampardi G , Mantia FL. Nat Commun 2022; 13: 687. 10.1038/s41467-022-28381-x 35115524 PMC8814157

[4] Liu C , Xie X, Lu B et al. ACS Energy Lett 2021; 6: 1015–33. 10.1021/acsenergylett.0c02684

[5] Ciurduc D E , Cruz CDL, Patil N et al. Energy Storage Mater 2022; 53: 532–43. 10.1016/j.ensm.2022.09.036

[6] Xie X , Li J, Xing Z et al. Natl Sci Rev 2023; 10: nwac281. 10.1093/nsr/nwac281 36875786 PMC9976762

